# Correction: A contemporary tool for assessing instrumental activities of daily living: Validation of a caregiver-reported scale for non-institutionalized older adults

**DOI:** 10.1371/journal.pone.0345869

**Published:** 2026-03-25

**Authors:** Zainab Barakat, Hala Sacre, Sarah Khatib, Aline Hajj, Carmela Bou Malham, Chadia Haddad, Rony M. Zeenny, Marwan Akel, Linda Abou Abbas, Marc Barakat, Samar Rachidi, Pascale Salameh

The fifth author’s name is spelled incorrectly. The correct name is: Carmela Bou Malham. The correct citation is: Barakat Z, Sacre H, Khatib S, Hajj A, Malham CB, Haddad C, et al. (2025) A contemporary tool for assessing instrumental activities of daily living: Validation of a caregiver-reported scale for non-institutionalized older adults. PLoS One 20(5): e0322554. https://doi.org/10.1371/journal.pone.0322554

An additional new affiliation is missing for the fifth author. Carmela Bou Malham is affiliated with the Maintain Aging Research Team, CERPOP, UMR 1295, University of Toulouse, INSERM, University of Toulouse III Paul Sabatier, Toulouse, France.

An additional new affiliation is missing for the sixth author. Chadia Haddad is affiliated with the Research Department, Psychiatric Hospital of the Cross, Jal Eddib, Lebanon and Faculty of Public Health, Lebanese University, Fanar, Lebanon
