# Supplementary figures and images for: A contemporary tool for assessing instrumental activities of daily living: Validation of a caregiver-reported scale for non-institutionalized older adults
Source: PLoS One. 2025 May 7;20(5):e0322554. doi: 10.1371/journal.pone.0322554 (PMC12057986; doi:10.1371/journal.pone.0322554)

## S1 File. Flowchart of the study phases


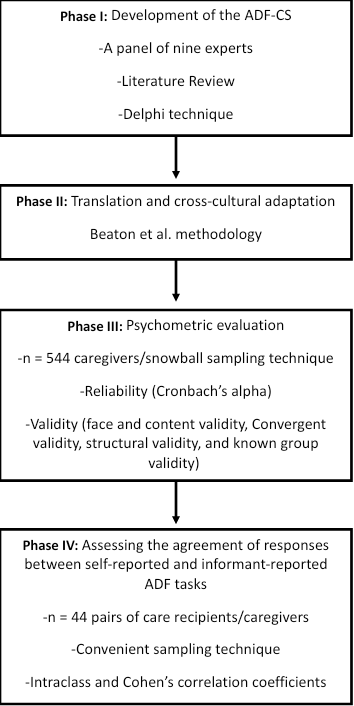

Supplement: S1 File — (DOCX) [file pone.0322554.s001.docx]
