# Supplementary material for: A contemporary tool for assessing instrumental activities of daily living: Validation of a caregiver-reported scale for non-institutionalized older adults
Source: PLoS One. 2025 May 7;20(5):e0322554. doi: 10.1371/journal.pone.0322554 (PMC12057986; doi:10.1371/journal.pone.0322554)
Supplement: S2 File — (DOCX) [file pone.0322554.s002.docx]

**S2 File. The ADF-CS English-Arabic version**

المقياس المعاصر لاستقلالية الأداء اليومي **(Autonomy in Daily Functioning-Contemporary Scale)**

| **أ. القدرة على استعمال الهاتف الثابت (Ability to use the landline telephone)** |
| --- |
| 1. يستعمل الهاتف باستقلالية فيبحث عن الرقم ويطلبه.  Uses the phone independently, searches for the number, and dials it  2. يطلب بعض الأرقام التي يعرفها.  Dials some number he/she knows  3. يجيب على الهاتف ولكن لا يطلب رقماً.  Answers the phone but does not dial a number  4. لا يستطيع إستعمال الهاتف على الإطلاق.  Cannot use the phone at all |
| **ب. القدرة على استعمال الهاتف المحمول (Ability to use a mobile phone)** |
| 1. يستعمل الهاتف المحمول باستقلالية، بما في ذلك البحث عن الأرقام والاتصال واستخدام واتس آب.  Uses the mobile phone independently, including searching for numbers, dialing, and using WhatsApp  2. يحتاج إلى بعض المساعدة: يطلب بعض الأرقام المحفوظه في الجوال.  Needs some help: Dials some numbers saved on his/her mobile phone  3. يجيب على الهاتف ولكن لا يطلب رقماً.  Answers the mobile phone but does not dial a number  4. لا يستطيع إستعمال الهاتف المحمول على الإطلاق.  Cannot use the mobile phone at all |
| **ت. القدرة على الذهاب إلى أماكن خارج مسافة المشي (Ability to go to places outside walking distance)** |
| 1. يستطيع التنقل باستقلالية (يقود سيارته الخاصة أو يستخدم وسائل النقل العام).  Travels independently on public transportation, or drives own car  2. يستطيع التنقل باستقلالية في التاكسي ولكن ليس في وسائل النقل العام.  Arranges own travel via taxi, but does not otherwise use public transportation  3. يستطيع استخدام النقل العام إذا كان يرافقه أحداً.  Travels on public transportation when assisted or accompanied by another  4. تقتصر تنقلاته على سيارة أجرة أو سيارة خاصة إذا ساعده شخص آخر.  Travel limited to taxi, automobile, or ambulette, with assistance  5. لا يتنقل أبداً.  Does not travel at all |
| **ث. القدرة على التسوّق (Shopping ability)** |
| 1. يتولى جميع احتياجات التسوق بشكل مستقل.  Takes care of all shopping needs independently  2. يتسوق بشكل مستقل للمشتريات الصغيرة.  Shops independently for small purchases  3. يحتاج إلى أن يكون مصحوباً في أي رحلة تسوق.  Needs to be accompanied on any shopping trip  4. غير قادر تماماً على التسوق.  Completely unable to shop |
| **ج. القدرة على التدبير المنزلي (Housekeeping ability)** |
| 1. يدبّر شؤون المنزل بمفرده أو بمساعدة من حين إلى آخر، مثلاً مساعدة منزلية في الأعمال الكبيرة.  Maintains house alone or with occasional assistance (e.g., heavy work done by domestic help)  2. يقوم بأعمال يومية خفيفة مثل غسل الأطباق وترتيب السرير.  Performs light daily tasks such as dishwashing and bed-making  3. يقوم بأعمال يومية خفيفة ولكن لا يمكنه الحفاظ على مستوى مقبول من النظافة.  Performs light daily tasks but cannot maintain an acceptable level of cleanliness  4. يحتاج إلى مساعدة في جميع الأعمال المنزلية.  Needs help with all home maintenance tasks  5. لا يشارك في أي من الأعمال المنزلية.  Does not participate in any housekeeping tasks |
| **ح. مسؤولية تناول الأدوية (Responsibility for own medication)** |
| 1. مسؤول عن تناول الدواء بالجرعات الصحيحة في الوقت الصحيح.  Is responsible for taking medication in correct dosages at the correct time  2. يتحمل المسؤولية إذا تم تحضير الدواء مسبقاً بجرعات منفصلة.  Takes responsibility if medication is prepared in advance, in separate dosages  3. غير قادر على تدبير أدويته.  Is not capable of dispensing own medication |
| **خ. القدرة على تحضير الطعام (Ability to prepare food)** |
| 1. يخطط ويحضّر ويقدم وجبات ملائمة بشكل مستقل.  Plans, prepares, and serves adequate meals independently  2. يحضّر وجبات ملائمة إذا تم تزويده بالمكونات.  Prepares adequate meals if supplied with ingredients  3. يسخن ويقدم ويحضّر الوجبات، أو يحضّر الوجبات، ولكنه لا يحافظ على نظام غذائي مناسب.  Heats and serves prepared meals, or prepares meals but does not maintain an adequate diet  4. يحتاج إلى أن تُحضَّر وتُقدَّم له وجبات الطعام.  Needs to have meals prepared and served |
| **د. القدرة على السفر لوحده (Solo travel abroad)** |
| 1. يستطيع السفر باستقلالية  Can travel independently  2. يستطيع السفر إذا رافقه شخص آخر  Can travel if accompanied by another person  3. غير قادر على السفر إلا إذا تم ترتيب طوارئ مثل استخدام مركبة متخصصة (كرسي متحرك)  Unable to travel unless an emergency evacuation has been arranged such as the use of a specialized vehicle (wheelchair) |
| **ذ. القدرة على استعمال التلفزيون (Ability to use television)** |
| 1. يستعمل التلفزيون بمفرده باستقلالية تامة.  He uses the TV alone with complete independence  2. يستطيع أن يبحث عن بعض القنوات المفضلة.  Can search for some favorite channels  3. لا يستطيع إستعمال التلفزيون على الإطلاق.  Cannot use the television at all |
| **ر. القدرة على إدارة الشؤون المالية (Ability to handle finances)** |
| 1. مستقل تماماً في إدارة الأمور المالية (الميزانيات، كتب الشيكات، دفع الإيجار، الفواتير، الذهاب إلى البنك)، يجمع ويتتبع الدخل.  Manages financial matters independently (budgets, writes checks, pays rent and bills, goes to bank); collects and keeps track of income  2. يدير المشتريات اليومية ولكنه يحتاج إلى المساعدة في الأعمال المصرفية والمشتريات الكبيرة والإنفاق الخاضع للرقابة، وما إلى ذلك  Manages day-to-day purchases but needs help with banking, major purchases, controlled spending, and so on  3. غير قادر على التعامل بالمال.  Incapable of handling money |
| ز. القدرة على استعمال الآلات الكهربائية المنزلية (غساله، نشافه، جلاية، مايكرويف، فرن كهرباء، مكنسه كهربائية)  **Ability to use household electrical appliances (washing machine, dryer, dishwasher, microwave, electrical oven, and vacuum cleaner)** |
| 1. يستطيع استعمال الأجهزة بدون مساعدة.  Can use electrical appliances without assistance  2. يحتاج إلى بعض المساعدة لتشغيل الأجهزة.  Needs some help to use electrical appliances  3. لا يستطيع استعمال الأجهزة الكهربائية على الإطلاق.  Cannot use electrical appliances at all  4. لم يستعمل هذه الأجهزة من قبل/ لا يعرف  Has never used these devices before / Don’t know |
