# Supplementary material for: A contemporary tool for assessing instrumental activities of daily living: Validation of a caregiver-reported scale for non-institutionalized older adults
Source: PLoS One. 2025 May 7;20(5):e0322554. doi: 10.1371/journal.pone.0322554 (PMC12057986; doi:10.1371/journal.pone.0322554)
Supplement: S3 File — (DOCX) [file pone.0322554.s003.docx]

## S3 File. Details of the five-step methodology utilized to translate the scales

Step 1 of the scale translation process involved two independent translators, native Arabic speakers, translating the scale from English to Arabic. The translators were instructed to avoid literal translation and use clear and culturally appropriate language for the Lebanese population. In Step 2, the two translators synthesized their translations, resolving any discrepancies between the initial translations through discussion and consensus to create a final Arabic version of the tool. Step 3 involved a translator conducting a back translation and comparing the back translation version with the original English version. In Step 4, an expert committee comprising specialists in geriatrics, public health, and epidemiology reviewed all translated versions. Each panel member evaluated the translations independently, focusing on linguistic, idiomatic, semantic, and cultural differences in each questionnaire item. Translation inconsistencies were resolved through consensus, leading to the development of a final scale version for field testing. Step 5 included administering the final version to a sample of 15 caregivers of older adults for pre-testing. The caregivers participated in written interviews to discuss the scale’s items’ meaning, comprehensibility, and acceptability.
