# Supplementary material for: A contemporary tool for assessing instrumental activities of daily living: Validation of a caregiver-reported scale for non-institutionalized older adults
Source: PLoS One. 2025 May 7;20(5):e0322554. doi: 10.1371/journal.pone.0322554 (PMC12057986; doi:10.1371/journal.pone.0322554)
Supplement: Attachment — Submitted filename: Response to Reviewers_ADF-CS Article.docx [file pone.0322554.s006.docx]

March 20, 2025

Dear Dr Maria José Nogueira,

We are pleased to resubmit the revised version of Manuscript entitled: “**A contemporary tool for assessing instrumental activities of daily living: validation of a caregiver-reported scale for non-institutionalized older adults.”**

**Ref:** Submission ID 36cd00b93930ffc3

We would like to thank the editor and the reviewer for their very helpful comments. We have carefully addressed all the comments. We hope our revision has improved the paper to a level of their satisfaction. Our responses are in italics and are prefaced by “Author response.” Corresponding changes are highlighted in the revised file. Please find attached our revised paper, and below is a summary of how we responded to the comments. My coauthor and I appreciate the opportunity to resubmit and are excited about the possibility of publication.

**Editor**

When submitting your revision, we need you to address these additional requirements.

**Thank you for your time and editorial guidance!**

1. Please ensure that your manuscript meets PLOS ONE's style requirements, including those for file naming. The PLOS ONE style templates can be found at

<https://journals.plos.org/plosone/s/file?id=wjVg/PLOSOne_formatting_sample_main_body.pdf> and <https://journals.plos.org/plosone/s/file?id=ba62/PLOSOne_formatting_sample_title_authors_affiliations.pdf>

**Author response:** *Thank you for your comment! I have reviewed the PLOS ONE style requirements, including those for file naming, and can confirm that the manuscript complies with these guidelines. Please let me know if any additional revisions are needed.*

2. In the online submission form, you indicated that Data are available from the corresponding authors upon reasonable request. All PLOS journals now require all data underlying the findings described in their manuscript to be freely available to other researchers, either a. In a public repository, b. Within the manuscript itself, or c. Uploaded as supplementary information.

This policy applies to all data except where public deposition would breach compliance with the protocol approved by your research ethics board. If your data cannot be made publicly available for ethical or legal reasons (e.g., public availability would compromise patient privacy), please explain your reasons on resubmission and your exemption request will be escalated for approval.

**Author’s response:** *Uploaded as supplementary information.*

3. Please review your reference list to ensure that it is complete and correct. If you have cited papers that have been retracted, please include the rationale for doing so in the manuscript text, or remove these references and replace them with relevant current references. Any changes to the reference list should be mentioned in the rebuttal letter that accompanies your revised manuscript. If you need to cite a retracted article, indicate the article’s retracted status in the References list and also include a citation and full reference for the retraction notice.

**Author’s response:** *Reviewed and verified. No retracted papers have been cited.*

**Reviewer**

I read your work entitled “A Contemporary Tool for Assessing Instrumental Activities of Daily Living: Validation of a Caregiver-Reported Scale for Non-Institutionalized Older Adults.”

**Thank you for taking time to review the article and for providing such valuable input!**

Here I enclose my recommendations to you:
1. There is a need for editing many English language errors. Please, have a more thorough “look” in the text by a native speaker of English or an editing office.

**Author response:** *Thank you for your comment! As requested, the English language errors have been corrected by a native English speaker as requested.*

2. The “Introduction” is poor and the rational of this study is not clear. The Authors have to address a lot in this work since they targeted to specific population. The translated versions of Lawton’s IADL are not all reported, is suggest the Authors to include them see a quick search I have performed for consultation (<https://scholar.google.com/scholar?hl=el&as_sdt=0%2C5&q=lawton%27s+iadl+scale+validation&btnG>= <https://scholar.google.com/scholar?hl=el&as_sdt=0%2C5&q=lawton%27s+iadl+scale+validation+greek&btnG>=).

**Author response:** *Thank you for pointing out these issues! I have revised the introduction to clearly express the rationale of the study. Additionally, I have reviewed and updated the references of the translated versions of the IADL Lawton as requested. Please refer to the revised manuscript for the updates. I would greatly appreciate any further suggestions or if you require additional modifications.*

3. The “Methods and the Results” are readers friendly and I congratulate the Authors for that.

**Author response:** *Thank you for your valuable feedback!*

4. The discussion also must be oriented to the population included in this study. If the Authors will updated their Introduction as suggested above they can enrich their discussion with more studies and strengthen more this section.

**Author response**: *Thank you for your comment! Our study developed a new scale, which makes direct comparisons with other studies challenging. However, we have made careful efforts to include relevant comparisons where possible. As requested, the discussion has been revised and encriched with additional studies. Please refer to the revised manuscript for the updates. I would greatly appreciate any further suggestions or if you require any additional modifications.*
